# Reading of small bowel capsule endoscopy after frame reduction using an artificial intelligence algorithm

**DOI:** 10.1186/s12876-024-03156-4

**Published:** 2024-02-22

**Authors:** Dong Jun Oh, Youngbae Hwang, Sang Hoon Kim, Ji Hyung Nam, Min Kyu Jung, Yun Jeong Lim

**Affiliations:** 1 Department of Internal Medicine, Dongguk University Ilsan Hospital, Dongguk University College of Medicine, 27 Dongguk-ro, Ilsandong-gu, Goyang, 10326 Republic of Korea; 2 Department of Electronics Engineering, Chungbuk National University, Cheongju, Republic of Korea; 3 Department of Internal Medicine, Chung-Ang University Gwangmyeong Hospital, Chung-Ang University College of Medicine, Gwangmyeong, Republic of Korea; 4 Division of Gastroenterology and Hepatology, Department of Internal Medicine, Kyungpook National University Hospital, Daegu, Republic of Korea

**Keywords:** Artificial intelligence, Capsule endoscopy, Frame reduction, Mucosal visualization

## Abstract

**Objectives:**

Poorly visualized images that appear during small bowel capsule endoscopy (SBCE) can confuse the interpretation of small bowel lesions and increase the physician’s workload. Using a validated artificial intelligence (AI) algorithm that can evaluate the mucosal visualization, we aimed to assess whether SBCE reading after the removal of poorly visualized images could affect the diagnosis of SBCE.

**Methods:**

A study was conducted to analyze 90 SBCE cases in which a small bowel examination was completed. Two experienced endoscopists alternately performed two types of readings. They used the AI algorithm to remove poorly visualized images for the frame reduction reading (AI user group) and conducted whole frame reading without AI (AI non-user group) for the same patient. A poorly visualized image was defined as an image with < 50% mucosal visualization. The study outcomes were diagnostic concordance and reading time between the two groups. The SBCE diagnosis was classified as Crohn’s disease, bleeding, polyp, angiodysplasia, and nonspecific finding.

**Results:**

The final SBCE diagnoses between the two groups showed statistically significant diagnostic concordance (k = 0.954, *p* < 0.001). The mean number of lesion images was 3008.5 ± 9964.9 in the AI non-user group and 1401.7 ± 4811.3 in the AI user group. There were no cases in which lesions were completely removed. Compared with the AI non-user group (120.9 min), the reading time was reduced by 35.6% in the AI user group (77.9 min).

**Conclusions:**

SBCE reading after reducing poorly visualized frames using the AI algorithm did not have a negative effect on the final diagnosis. SBCE reading method integrated with frame reduction and mucosal visualization evaluation will help improve AI-assisted SBCE interpretation.

## Introduction

Small bowel capsule endoscopy (SBCE) is the primary process in the diagnosis of various small bowel (SB) diseases, such as suspected obscure gastrointestinal (GI) bleeding, Crohn’s disease, SB tumor or polyposis, and celiac disease [1–3]. SBCE is patient-friendly, but burdensome to physicians because of its long reading time [4]. Also, because SBCE cannot cleanse the mucosa, poorly mucosal visualized images inevitably appear during SBCE reading. These poorly visualized images not only interfere with the accurate interpretation of SB lesions, but may also lead to re-examination [5]. A previous meta-analysis reported that the rate of well visualized images was only 49% when bowel preparation is not performed for SBCE [6]. Even with bowel preparation, poor visualization (mucosal visualization < 50%) was reported in about 13% of the cases [7].

However, the physician cannot manually and accurately skip only poorly visualized images that appear unexpectedly during the whole SBCE frame. Focusing on poorly visualized images will exhaust the physician. Poor image quality such as opacity, blurriness, and contrast can also negatively affect lesion detection [8]. If poorly visualized images can be removed using an artificial intelligence (AI) algorithm, it can reduce the number of SBCE frames to be read and reduce the burden of SBCE reading. Furthermore, if only clearly visualized images are inspected, the SBCE reading can be done quickly and efficiently.

Recent studies have developed AI algorithms that can automatically measure the SB mucosal visualization score (1: very poor to 5: excellent) [9, 10]. This study aimed to compare the diagnostic concordance in SBCE diagnosis between whole SBCE frame reading as a conventional method and frame reduction reading after automatically removing poorly visualized images using this AI algorithm.

## Patients and methods

### Study data source and variables

Anonymized SBCE (MiroCam, Intromedic Co., Ltd., Korea) cases from other institutions from January 2020 to December 2020 were used in this study. Ninety-nine cases of SBCE were identified, and incomplete studies in which cecal transit was not confirmed were excluded. Ninety SBCE cases were finally enrolled, which were not used and involved in a previous AI algorithm study.

Based on the electronic medical records and SBCE images, the following variables were analyzed: reason for SBCE, SB transit time, SBCE reading time, SBCE lesion (inflamed lesion, hemorrhagic lesion, polypoid lesion, vascular lesion, and nonspecific lesion such as lymphangiectasia, diverticulum) [11], SBCE diagnosis [Crohn’s disease, bleeding, polyp, angiodysplasia (including angio-ectasia), and nonspecific finding], and mucosal visualization score measured by the AI algorithm. Informed consent for study participation was obtained from all the subjects. This study protocol was conducted in accordance with the guidelines of the Declaration of Helsinki. It was approved by the Institutional Review Board of the Dongguk University Ilsan Hospital (DUIH IRB No. 2018-10-009).

### Frame reduction of poorly visualized images using AI algorithm

In this study, a validated AI algorithm was applied to calculate the visualization score of each SBCE image using a 5-step scoring method: score 1, very poor (mucosal visualization < 25%); score 2, poor (mucosal visualization 25–49%); score 3, fair (mucosal visualization 50–74%); score 4, good (mucosal visualization 75–89%); and score 5, excellent (mucosal visualization > 90%) [9, 10]. Using this AI algorithm, the mucosal visualization scores for each image and case were derived. Subsequently, very poor and poor visualization images, defined as poorly visualized images, from whole images were removed, and the remaining SBCE images were saved separately.

### SBCE reading methods with the whole frame and frame reduction reading

**Fig. 1:**
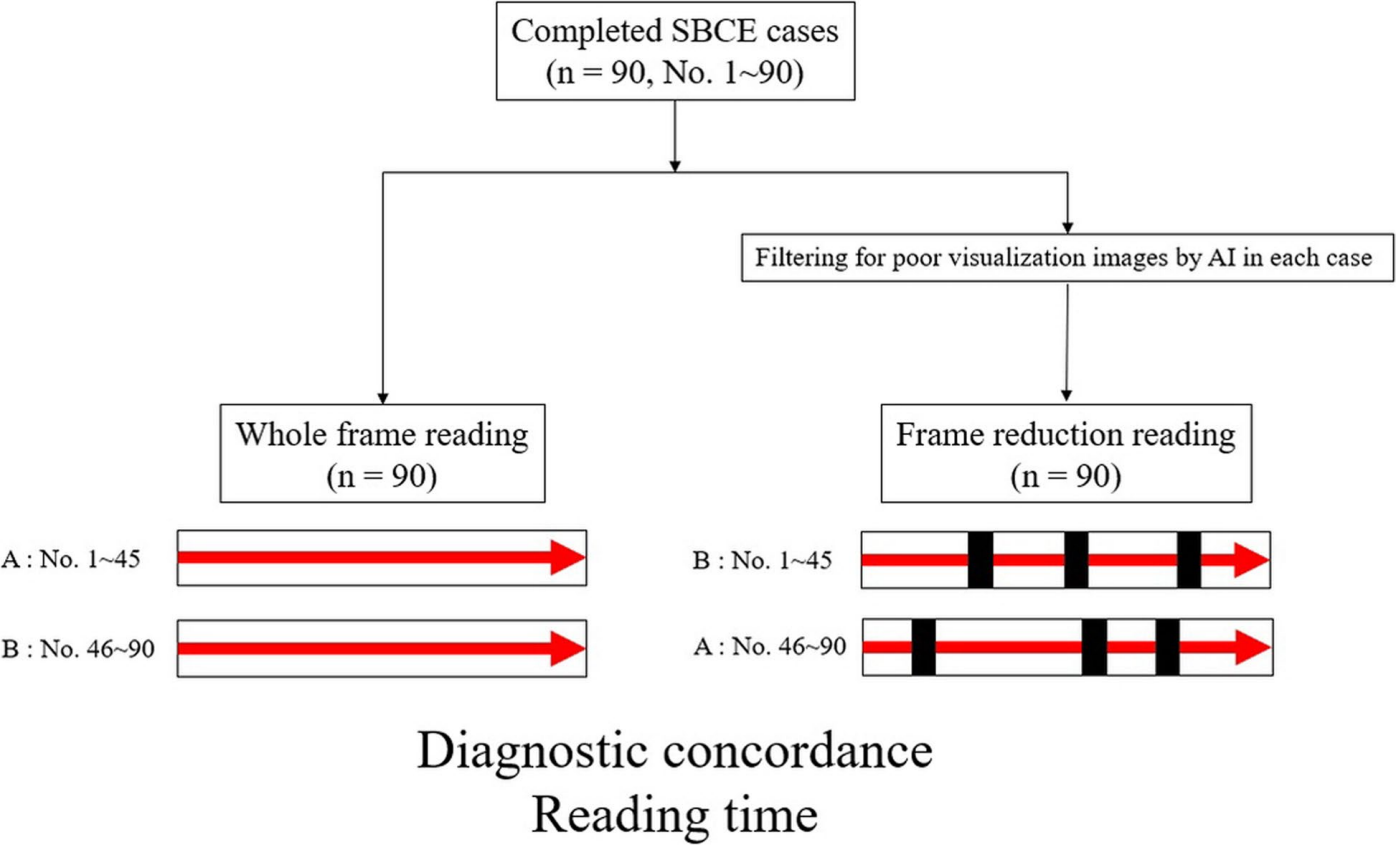
Schematic diagram of this study. Expert endoscopists A and B each performed readings for half of SBCE cases with whole frame reading and artificial intelligence (AI) filtered frame reduction reading

Two expert endoscopists (Oh DJ and Kim SH) performed the SBCE reading in a matching study. Ninety SBCE cases were divided into two halves (nos. 1–45 and nos. 46–90). Two endoscopists performed whole frame reading (AI non-user group) and frame-reduction reading (AI user group) (Fig. 1). For example, for SBCE cases of nos. 1–45, Oh DJ performed whole frame readings and Kim SH performed frame reduction readings. Conversely, for SBCE cases of nos. 46–90, Kim SH performed whole frame readings and Oh DJ performed frame reduction readings. Immediately after the SBCE reading, the results of the two endoscopists were sent directly to an external endoscopist (Nam JH, Jung MK) for analysis.

#### Whole frame reading (AI non-user group)

Two endoscopists read the whole frame SBCE images manually using a software viewer (MiroView, Intromedic Co., Ltd., Korea). According to the guideline, the reading speed was less than 10 frames per second in a single view [4]. The SBCE lesions identified during reading were captured and annotated separately using the software viewer. The reading time and final SBCE diagnosis were determined after the SBCE reading.

#### Frame reduction reading (AI user group)

Two endoscopists also performed SBCE reading using the frame reduction method. First, the AI ​​algorithm calculated the mucosal visualization score for each SBCE image. Then, poorly visualized images with scores of less than three were removed. The remaining SBCE images were separately saved by AI researcher (Hwang Y) and uploaded to the software viewer. Subsequently, the SBCE images with the reduced frames was read using a software viewer. The reading process was the same as the aforementioned whole frame reading.

### The calculation of the mucosal visualization score in SBCE

In this study, we measured and compared the mucosal visualization scores in both the AI user and non-user groups. In the AI user group, the AI algorithm automatically calculated the scores for each image in the SBCE using a 5-step scoring method from 1 (very poor) to 5 (excellent). Subsequently, the scores for each image were summed and averaged to determine the visualization score of the SBCE. In the AI non-user group, the mucosal visualization scores were manually calculated using a 5-step scoring method. Similar to the conventional method widely used for the visualization score scale [2], in this study, we evaluated the scores of images at 5-minute intervals using the 5-step scoring method from score 1 (very poor) to 5 (excellent). The average of scores at 5-minute intervals was used to determine the visualization score of the case.

### Outcomes and statistical analyses

The primary outcome was to determine whether there was a difference in diagnosis between whole frame reading and frame reduction reading. Secondary outcomes were the ratio of poorly visualized images excluded by the AI algorithm from the whole SBCE frame, the number of lesion images included in poorly visualized images, and the difference in reading time between the AI non-user and the AI user groups. Student’s t-test and Chi-square test were used to analyze the variables. The Intraclass Correlation Coefficient was used to measure the diagnostic concordance between the two groups. Statistical significance was set at *p* < 0.05 in both univariate analyses. All statistical analyses were performed using IBM SPSS Statistics v25.

## Results

### Baseline characteristics and number of SBCE images

Reasons for SBCE were suspected Crohn’s disease in 43 cases (47.8%), SB bleeding in 30 cases (33.3%), SB polyp in 14 cases (15.6%), and chronic diarrhea in 3 cases (3.3%). The mean SB transit time was 287.0 ± 121.3 min. In the AI non-user group, the mean number of the entire SB images was 46,627.7 ± 20,684.7 (range 7,178 − 106,866). In the AI user group, the mean number of the remaining SB images was 27,711.3 ± 18,145.7 (range 331 − 92,877).

### Mucosal visualization score calculated by the AI algorithm

Poorly visualized images identified by the AI ​​algorithm were 40.6% of all SBCE images (Table 1). There was one case in which 99% (32,613 out of 32,994 images) of the SBCE frames was removed by the AI algorithm. This case was confirmed to have overall poorly visualized images due to active bleeding. The mean overall mucosal visualization score of the AI user group measured by the AI ​​algorithm was 3.36 ± 1.0. In each case, the mean score difference between the AI non-user group and the AI user group was 0.29 ± 0.88 (range 0.02–2.45). When the overall visualization score was classified for each case, very poor, poor, fair, good, and excellent results were identified in 1, 22, 23, 33, and 11 cases, respectively.

**Table 1:** Baseline characteristics of small bowel capsule endoscopy (SBCE) cases

Variables	*n* = 90
Reasons for SBCE	
Suspected small bowel Crohn’s disease	43 (47.8%)
Suspected small bowel bleeding	30 (33.3%)
Suspected small bowel polyp	14 (15.6%)
Others^a^	3 (3.3%)
Mean small bowel transit time	287.0 ± 121.3 mins
Mean number of images in full length of small bowel	46627.7 ± 20684.7
Bowel cleanliness measured by artificial intelligence	
Mean visualization score	3.36 ± 1.0
Very poor	1 (1.1%)
Poor	22 (24.4%)
Fair	23 (25.6%)
Good	33 (36.7%)
Excellent	11 (12.2%)

^a^Chronic diarrhea

### Diagnostic concordance between the AI user and non-user groups

**Fig. 2:**
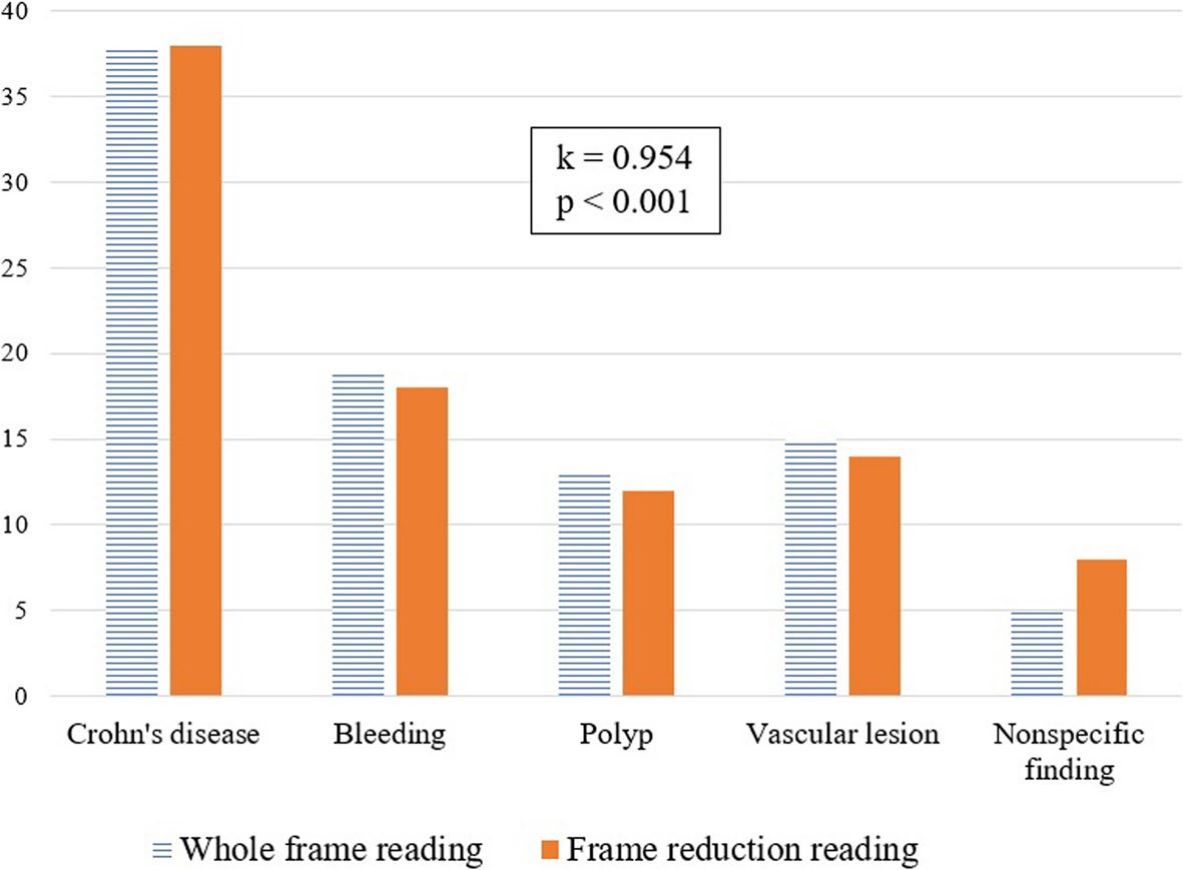
Comparison of diagnosis of whole frame reading and frame reduction reading. The diagnosis according to each case was significantly consistent between the two groups

In the AI non-user group, the final SBCE diagnoses were Crohn’s disease in 38 cases (42.2%), bleeding in 19 cases (21.1%), polyp in 13 cases (14.4%), angiodysplasia in 15 cases (16.7%), and nonspecific finding in 5 cases (5.6%). In the AI user group, the final SBCE diagnoses were Crohn’s disease in 38 cases (42.2%), bleeding in 18 cases (20.0%), polyp in 12 cases (13.3%), angiodysplasia in 14 cases (15.6%), and nonspecific finding in 8 cases (8.9%). When the diagnoses of the two groups were compared for each case, the diagnostic concordance was statistically significant (k = 0.954, *p* < 0.001). The diagnoses were different between the two groups in only three cases (Fig. 2). When analyzing the three cases in which the diagnosis differed between the two groups, the AI user group was diagnosed with nonspecific finding, whereas the AI non-user group was diagnosed with bleeding, polyp, and angiodysplasia.

### Lesion images between the AI user and non-user groups

The lesion image is defined as an image that includes inflamed lesion, hemorrhagic lesion, polypoid lesion, vascular lesion, and nonspecific lesion. The mean number of lesion images per case was 3008.5 ± 9664.9 (range 4-70512) in the AI non-user group and 1401.7 ± 4811.3 (range 2-32327) in the AI user group. In this study, we did not observe any cases in which all lesion images were removed in the AI user group. The mean number of lesion images in the AI non-user group was 808.3 ± 1339.6 for inflamed lesion, 11265.6 ± 19730.6 for hemorrhagic lesion, 1661.0 ± 2939.7 for polypoid lesion, 216.1 ± 345.6 for vascular lesion, and 234.4 ± 255.6 for nonspecific lesion. The mean number of lesion images in the AI user group was 414.6 ± 587.5 for inflamed lesion, 5213.1 ± 9647.4 for hemorrhagic lesion, 582.0 ± 1273.8 for polypoid lesion, 194.0 ± 321.6 for vascular lesion, and 175.6 ± 181.6 for nonspecific lesion. When AI algorithm was applied, the removal rates of lesion images for inflamed lesion, hemorrhagic lesion, polypoid lesion, vascular lesion, and nonspecific lesion were 48.7%, 53.7%, 65.0%, 10.2%, and 25.1%, respectively (Table 2). The total number of removed lesion images was 144,612, consisting 7,367 inflamed lesion, 112,892 hemorrhagic lesion, 7,635 polypoid lesion, 529 vascular lesion, and 16,189 nonspecific lesion.

**Table 2:** Comparison of changes in the whole frame reading and frame reduction reading after artificial intelligence filtration

Variable	Reading method		Remark
	Whole frame (*n* = 90)	Frame reduction (*n* = 90)	
Mean number of total images (range)	46627.7 ± 20684.7	27711.3 ± 18145.7 (331 ~ 92,877)	40.6%^a^
Mean number of lesion images (range)	3008.5 ± 9964.9 (4 ~ 70,512)	1401.7 ± 4811.3 (2 ~ 32,327)	53.4%^a^
inflamed lesion (*n* = 38)	808.3 ± 1339.6 (11.3%)	414.6 ± 587.5 (12.5%)	48.7%^a^
hemorrhagic lesion (*n* = 19)	11265.6 ± 19730.6 (79.1%)	5213.1 ± 9647.4 (78.5%)	53.7%^a^
polypoid lesion (*n* = 13)	1661.0 ± 2939.7 (8.0%)	582.0 ± 1273.8 (6.0%)	65.0%^a^
vascular lesion (*n* = 15)	216.1 ± 345.6 (1.2%)	194.0 ± 321.6 (2.3%)	10.2%^a^
nonspecific lesion (*n* = 5)	234.4 ± 255.6 (0.4%)	175.6 ± 181.6 (0.7%)	25.1%^a^
Mean reading times, mins (range)	120.9 ± 45.1 (25 ~ 236)	77.9 ± 49.4 (9 ~ 258)	*P* < 0.001

^a^Removal rate

### SBCE reading times

The SBCE reading was conducted using the same software viewer for both groups. The mean reading time was 120.9 ± 45.1 min (range 25–236 min) for the AI non-user group and 77.9 ± 49.4 min (range 9-258 min) for the AI user group, resulting in the reading time significantly reduced by 35.6% in the AI user group (*p* < 0.001).

## Discussion

This study was conducted to determine the efficiency and accuracy of SBCE reading after removing poorly visualized images (mucosal visualization score < 3) using AI. We identified that frame reduction reading after removing poorly visualized images using the AI ​​algorithm significantly reduced reading time without affecting the final SBCE diagnosis when compared to whole frame reading. Regarding the overall mucosal visualization score, the mean score difference between the AI ​​measurement and the physician measurement for each case was 0.29 ± 0.88, showing no significant difference.

In this study, when poorly visualized images were removed using AI in each case, a mean of 40.6% of the total SBCE images were removed. Also, when removing poorly visualized images, a mean of 53.4% ​​of total lesion images were removed. However, the diagnostic yield was maintained because there were no cases in which the lesions were completely removed. SBCE records images at a rate of two frames per second for 8–12 h [3]. Although it was difficult to detect SB lesions in poorly visualized images, there were cases in which disease was diagnosed by detecting the lesions in other adequately visualized images. In particular, in cases of polypoid or inflamed lesions, additional lesion images may be obtained because lesions can slow the passage of the capsule.

When comparing the AI non-user and user groups, the removal rates of images according to lesion were 48.7% for inflamed lesion, 53.7% for hemorrhagic lesion, 65.0% for polypoid lesion, 10.2% for vascular lesion, and 25.1% for nonspecific lesion. Most (112,892 out of 144,612, 78.1%) of the removed lesion images were hemorrhagic lesion. Because active bleeding and/or a large amount of blood clots often covered the mucosa, the AI ​​algorithm recognized them as poorly visualized images, leading to their removal. Also, the removal rate of the polypoid lesion images was higher than the mean removal rate (53.4%) of total lesion images. Polypoid lesions were often found in the distal small bowel, where mucosal visualization was often poor. As a result, polypoid lesion images were thought to have been removed more than other lesion images.

Because a mean of 40.6% of the SBCE images were removed in each case, the reading time in the frame reduction reading was also significantly shorter than that in the whole frame reading. This study was noteworthy in that both the AI non-user and AI user group were read using the same commercially available software viewer. A recent trend in SBCE reading using AI algorithm is computer-assisted lesion detection. Installing an AI algorithm in a software viewer is necessary to use AI in clinical practice [12]. In a previous study on using AI for detecting lesions in SBCE, it was not possible to reproduce the same SBCE reading as in real clinical practice because the AI non-user and AI user groups were read in different ways [13]. However, in this study, the SBCE reading was performed using the same software viewer, even after AI processing.

In our study, it was possible to calculate the SBCE mucosal visualization score using an AI algorithm. The mean difference between mucosal visualization scores measured by physicians and the AI ​​algorithm was similar with a difference of 0.29 ± 0.88. In most cases, the differences in scores between physicians and AI ​​algorithm were less than one point in each case. However, a difference of more than two points was confirmed in four cases. In all cases, the physicians judged the visualization score to be lower than the AI. In the AI non-user group, subjective judgment and inter-observer variation should be considered because the physician measured the visualization score simultaneously with the SBCE reading [14]. The guideline recommends maintaining adequate bowel preparation in more than 95% of elective SBCE [5]. The purpose of bowel preparation for SBCE was to read images more accurately by reducing poor visualization. However, there were still controversies about the optimal bowel preparation method, type, and time for SBCE [5, 15–17]. According to a recent randomized controlled trial, bowel preparation before SBCE did not improve the diagnostic yield and mucosal visualization compared to clear fluids only [18]. Although manual and AI calculation methods have been proposed for measuring bowel preparation quality [5, 19], there is no clear consensus regarding the measurement of SB cleanliness. Therefore, further studies using the AI algorithm are needed to investigate the effect of bowel preparation on full-length SBCE video and the consistency of bowel preparation scores with expert endoscopists.

**Fig. 3:**
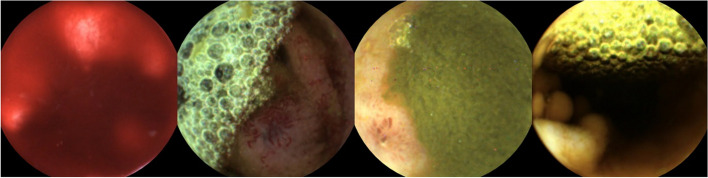
Examples of lesions removed while filtering out the poorly visualized images. Visibility of the mucosa was not good, but bleeding, vascular lesion, inflamed lesion and polypoid lesions were observed from left to right

Although the results of our study were good, we considered it challenging to apply this frame reduction method into real clinical practice. This study had several limitations. The most important limitation is the possibility that AI can erase meaningful frames containing significant lesions (Fig. 3). Significant lesions obscured by blood and intestinal debris may be missed. Of course, in this study, there was no difference in diagnostic accuracy because lesions were found in other images; however, errors can occur when using the frame reduction reading method alone. In this study, 3 cases had diagnostic differences between the two examiners. One polyp, one bleeding, and one angiodysplasia were misdiagnosed in the AI user group. When the AI removed poorly visualized images, significant lesions were also removed, and the AI ​​user group diagnosed these cases as nonspecific finding. Therefore, frame reduction reading should be used as an adjunct to AI algorithms integrated with lesion detection reading. For example, after lesion detection using AI, poorly visualized images can be removed by frame reduction to provide sharp and clear images. Second, there were few participating endoscopists and SBCE cases in our study because this study was conducted at a single center. The effectiveness of frame reduction reading should be confirmed in a multicenter, large-scale study in the future. Third, the MiroCam used in this study has not been approved in some regions and has a small market share worldwide. Therefore, further studies on other types of SBCEs will also be needed. Finally, in the frame reduction reading of this study, it was necessary to remove poorly visualized images and subsequently save and upload the remaining images. Therefore, there is a need for the development of software capable of real-time removal of poorly visualized images and merging the remaining images during SBCE reading.

## Conclusions

This study demonstrates the possibility of utilizing AI frame reduction methods, other than automatic lesion detection, for SBCE reading. Despite several limitations, our results will help expand AI-assisted SBCE readings in various multi-tasking ways and settle them into clinical practice [20]. Frame reduction reading, which automatically removes poorly visualized images using an AI algorithm, can shorten reading time without affecting the final SBCE reading. AI-assisted lesion detection is essential for physicians who read SBCE. However, additional studies are also needed to fully believe the results of SBCE reading using AI-assisted lesion detection alone. Frame reduction reading may serve as a bridge between conventional and AI-assisted reading.

## Data Availability

The datasets generated and/or analyzed during the current study are not publicly available because national projects related to companies but are available from the corresponding author upon reasonable request.

## Abbreviations

AI: Artificial intelligence
SBCE: Small bowel capsule endoscopy
GI: Gastrointestinal
